# Icilio Guareschi and his amazing “1897 reaction”

**DOI:** 10.3762/bjoc.17.93

**Published:** 2021-05-25

**Authors:** Gian Cesare Tron, Alberto Minassi, Giovanni Sorba, Mara Fausone, Giovanni Appendino

**Affiliations:** 1 Dipartimento di Scienze del Farmaco, Università degli Studi del Piemonte Orientale, Largo Donegani 2, 28100 Novara, Italy; 2 Sistema Museale di Ateneo, Archivio Scientifico e Tecnologico dell'Università di Torino, C.so Massimo D'Azeglio 52, 10126 Torino, Italy

**Keywords:** Guareschi, history of chemistry, hydrocarbons, name reactions, pyridine

## Abstract

Organic chemistry honors Icilio Guareschi (1847–1918) with three eponymic reactions, the best known ones being the Guareschi synthesis of pyridones and the Guareschi–Lustgarten reaction. A third Guareschi reaction, the so-called “Guareschi 1897 reaction”, is one of the most unusual reactions in organic chemistry, involving the radical-mediated paradoxical aerobic generation of hydrocarbons in near-neutral water solution. A discussion of the mechanism of this amazing reaction, the only metal-free process that generates hydrocarbons, and the implications of the approach in biology and geosciences mirrors the multifaceted scientific personality of the discoverer. Thus, Guareschi’s eclectic range of activities spans a surprising variety of topics, overcoming the boundaries of the traditional partition of chemistry into organic, inorganic, and analytical branches and systematically crosses the divide between pure and applied science as well as between the history of chemistry and the personal contributions to its development.

## Introduction

**Figure 1:**
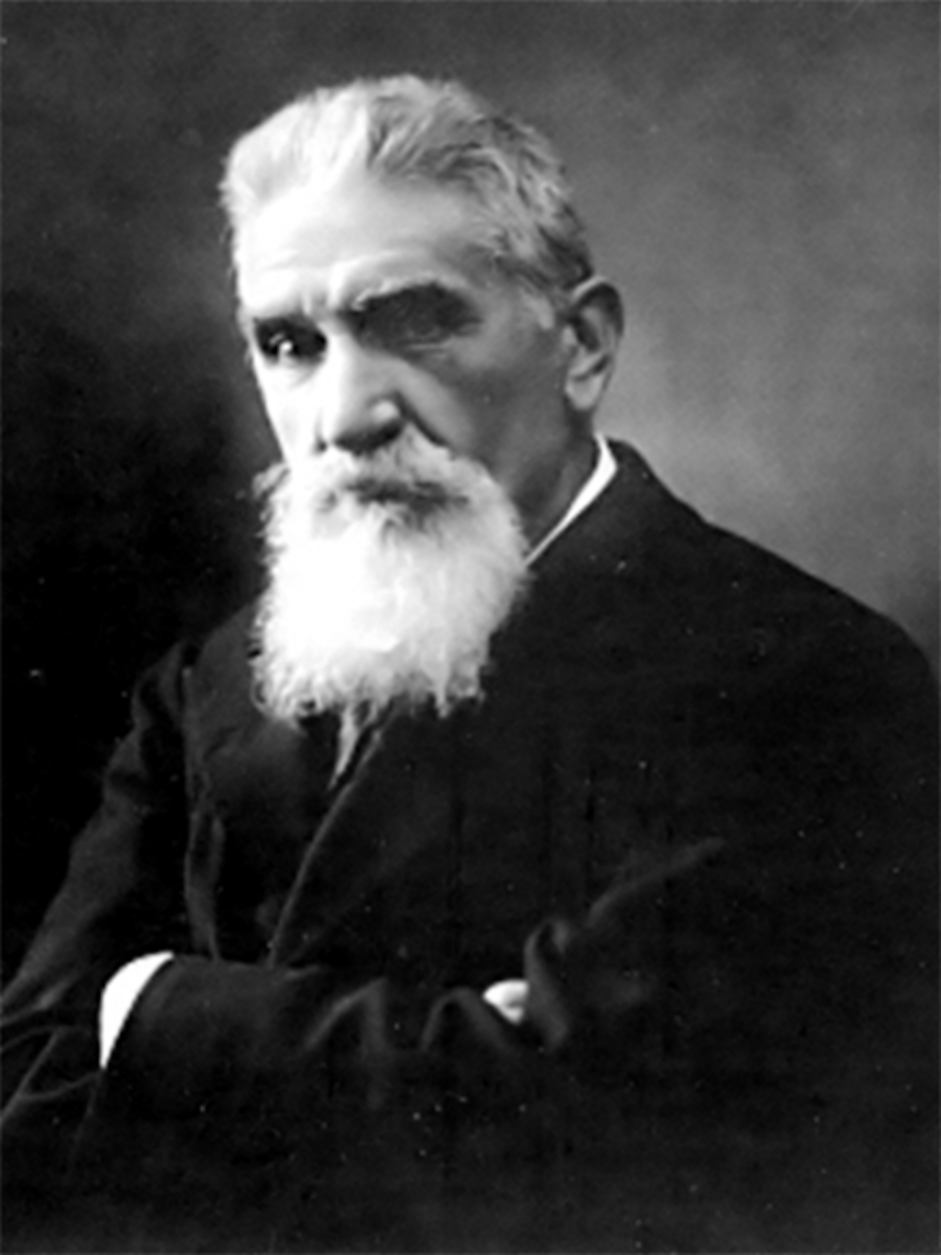
Icilio Guareschi (1847–1918). (Source: *Annali della Reale Accademia di Agricoltura di Torino* **1919**, *LXII*, 92). This content is not subject to CC-BY 4.0.

Modern science emphasizes focusing and prizes specialists over generalists. Nowadays, scientists "feed on one plant only", but their ancestors had a more varied diet. Zonation occurred rapidly in chemistry during the first decades of the 20th century, and Hammett, in the introduction of his Physical Organic Chemistry textbook, bemoaned already in 1940 that physical chemists and organic chemists proudly boasted their ignorance in one another’s discipline, with physicists considering the study of organic reactions “soapmaking” [1], a technical improvement from the status of “stamp collecting”, where Rutherford is rumored to have relegated chemistry. Icilio Guareschi (Figure 1, 1847–1918) belongs to the last generation of generalists, leaving his mark in different fields of chemistry and at the intersection with other sciences as well as with technology.

**Scheme 1:**
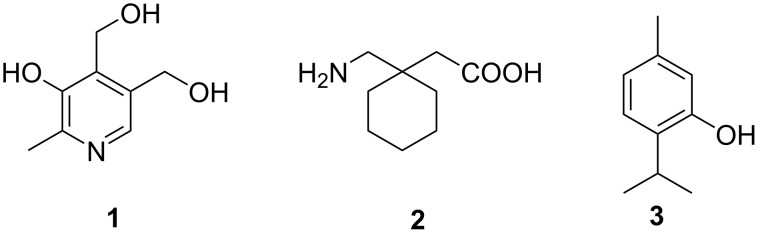
Vitamin B_6_ (pyridoxine, **1**), gabapentin (**2**), and thymol (**3**).

Organic chemistry honors Guareschi with three eponymous reactions. His synthesis of cyanopyridones was at the basis of the first industrial synthesis of pyridoxine (vitamin B_6_, **1**, Scheme 1) by Merck [2], is used for the preparation of the blockbuster drug gabapentin (**2**) [3], and features in countless medicinal chemistry projects based on the pyridine scaffold [4]. The Guareschi–Lustgarten reaction is at the basis of the pharmacopoeia assay of thymol (**3**) [5], and investigation on the color associated with the reaction eventually led to the discovery of the Reimer–Tiemann formylation of phenols [6]. Conversely, the third Guareschi eponymic reaction, the so-called “1897 reaction” is not well known [7], despite that the unique mechanistic aspects make it one of the most unusual reactions in organic chemistry, involving the paradoxical radical-mediated aerobic generation of hydrocarbons in water under only slightly basic conditions [8]. Also unappreciated are the implications of the “1897 reaction” outside chemistry and especially in biology and geosciences. These were already foreseen by Guareschi himself [7] but have surprisingly remained overlooked in the scientific literature, just like his “1897 reaction” remained forgotten or was even considered a blunder of the discoverer, until the clarification of the unique mechanism over a century after the original report [8].

Synthesis represents, however, only one facet of the polyhedral scientific personality of Icilio Guareschi, whose eclectic range of activities spans a surprising variety of topics, overcoming not only the boundaries of the traditional partition of chemistry in the organic, inorganic, and analytical branches but also crossing the divide between pure and applied science and between the history of chemistry and the contribution to its development. Guareschi, just like Liebig, seems to have cultivated an interest in every topic where a chemical connection could be identified, from geology to botany and from medicine to agriculture and nutrition. There is, undoubtedly, no shortage of eclectic characters in the history of chemistry, but the combination of interests for pure and applied science and erudition combine to make Guareschi a rare, if not unique figure, versatile to the point of including in his scientific activity fields as diverse as the discovery of new reactions and the restauration of parchments, and addressing, as an historian, topics as varied as the analysis of the Chinese contributions to chemistry, the elucidation of the genesis of the gas laws, and the Renaissance ink technology. Like many chemists of his time, his sincere pacifism was animated by an intense patriotism that, paradoxically, eventually led him to play an active role in WWI, developing the soda lime gas mask. The Guareschi mask was, regrettably, never used by the Italian army but predated by two years the development of the efficient gas masks used by the British and American soldiers in the last years of the war, deserving to the pacifist Guareschi a debatable place also in the history of chemical warfare [9].

## The early years (1847–1879): The foundation of the Gazzetta Chimica Italiana and the Guareschi–Lustgarten reaction

**Figure 2:**
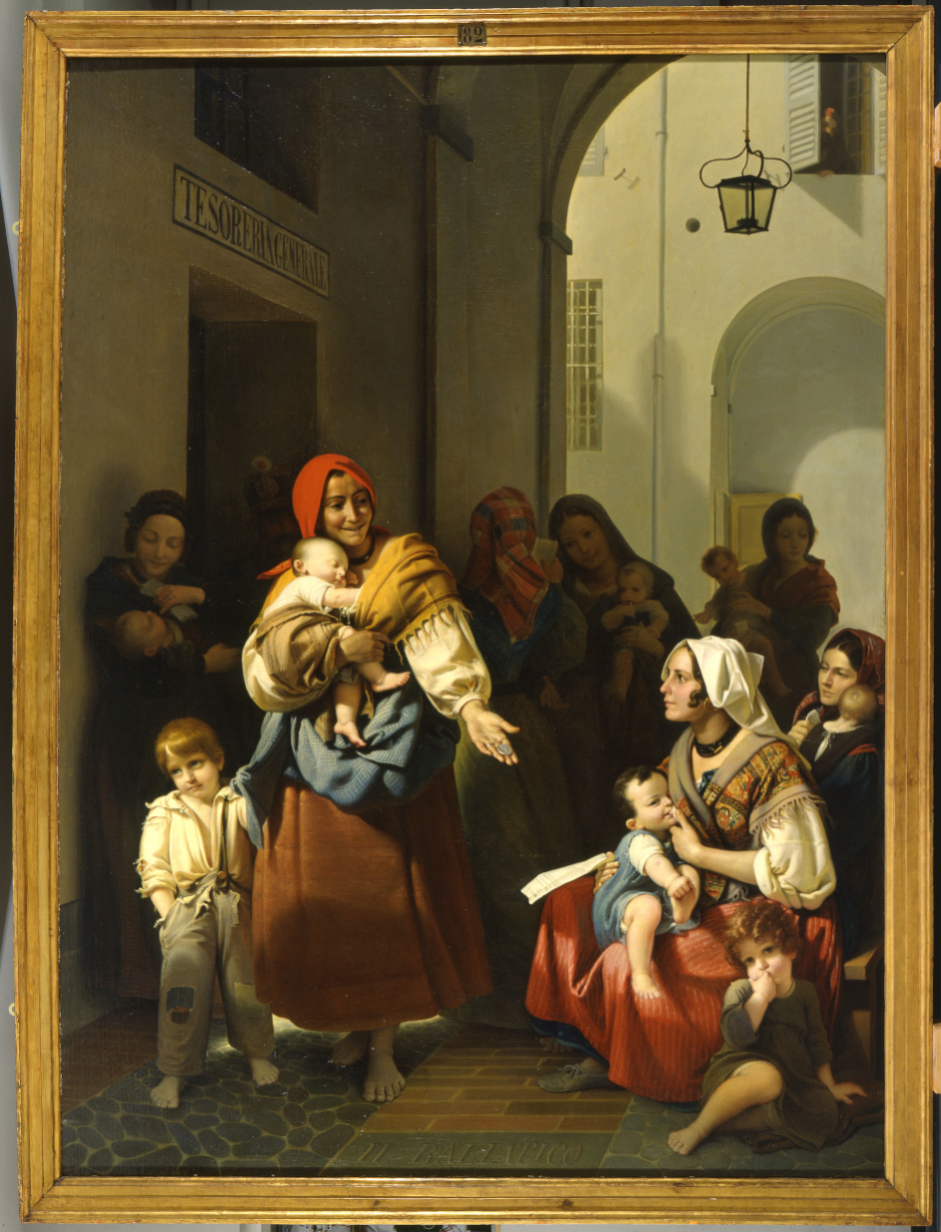
Baliatico (Nursing) by Francesco Scaramuzza (275 cm × 214 cm, Parma, Complesso Museale della Pilotta, Galleria Nazionale). Four-year-old Guareschi served as a model for the white-dressed blonde child on the left side of the painting. Image reused with permission by Ministero della Cultura – Complesso Monumentale della Pilotta – Galleria Nazionale (“su concessione del Ministero della Cultura – Complesso Monumentale della Pilotta – Galleria Nazionale”); image must not be reused without permission from the rights holders; this content is not subject to CC-BY 4.0.

Icilio Guareschi was born on Christmas Eve of 1847 in San Secondo, a small town near Parma, in Emilia (Northern Italy). San Secondo is famous for the connection to the painter Parmigianino, who lived there in 1538–1539 at the court of the count Pier Maria Rossi, immortalized with his wife Camilla Gonzaga and their three sons in two celebrated portraits now at the Prado Museum in Madrid [10–11]. In 1847, San Secondo was part of the Duchy of Parma, a small state created in 1814 to host Napoleon’s wife Marie Louise, who, incidentally, died the very same year Guareschi was born. Guareschi’s father was a pharmacist. The family pharmacy had been established in the early 1400 and had then been passed from father to son during four centuries. Giovannino Guareschi (1908–1968), the creator of the Don Camillo saga, belonged to a related but distinct branch of the family. Icilio’s mother (Francesca Scaramuzza) was a cousin of the painter Francesco Scaramuzza (1803–1886), famous for his illustrations of the Divina Commedia by Dante Alighieri. Guareschi grew up in a wealthy family, and when four years old, he even served as a model for a painting by Scaramuzza (Figure 2).

Guareschi claimed his early life was devoid of significant events [12], but in 1866, when 19 years old and still at the Liceo (the Italian High School) in Parma, he volunteered during the III Italian Independence War against the Austro–Hungarian Empire. Guareschi participated in the Borgoforte battle of July 18, one of the few victories of the Italian army in this strange war, lost by Italy at land (Custoza) and at sea (Lissa) but nevertheless yielding Venice as a results of the striking victories by the allied Prussia. Guareschi returned to school as a sergeant and had then to cope, just like Giovannino Guareschi several decades later, with the financial downturn of his family, an event triggered by the death of his eldest brother, who owned the family pharmacy. According to the Italian law at that time, if none in the family was a registered pharmacist or studying to get this title, the Pharmacy had to be publicly auctioned. Therefore, Icilio, who had planned to become an engineer or, as a second choice, to study economy [12], enrolled in the School of Pharmacy in Bologna in 1867, obtaining his Diploma three years later. In those times, Pharmacy Schools only granted a technical diploma, and a Laurea (Degree) in Pharmacy and Chemistry (Laurea in Chimica e Farmacia) was only established in 1874 at a few selected universities [13]. With the family’s pharmacy saved, he could then continue his studies at the University of Pisa, attending the laboratory of Hugo Schiff (1834–1915) in Florence and eventually graduate in Natural Sciences in 1871 at the University of Pisa. The University of Florence was only established in 1924, but the town hosted several scientific institutions, one of the most remarkable ones being the Museo di Scienze Naturali, where in March 1814, Humphry Davy had made his experiment on the combustion of a diamond. Hugo Schiff had been teaching and doing research there since 1864, called from Pisa with the support of his elder brother, the prominent physiologist Moritz Schiff (1823–1892), who was already teaching in Florence [14]. After graduation, Guareschi returned to the University of Bologna as a lecturer of analytical chemistry in the group of Francesco Selmi (1817–1881), one of the founders of colloid chemistry and the initiator of the studies on ptomaines, the nitrogenous compounds produced in decaying corpses (see infra). Selmi supported additional stays of Guareschi in Schiff’s laboratory up to 1873, when Guareschi obtained the Chair of Chemistry at the Technical High School of Livorno.

**Figure 3:**
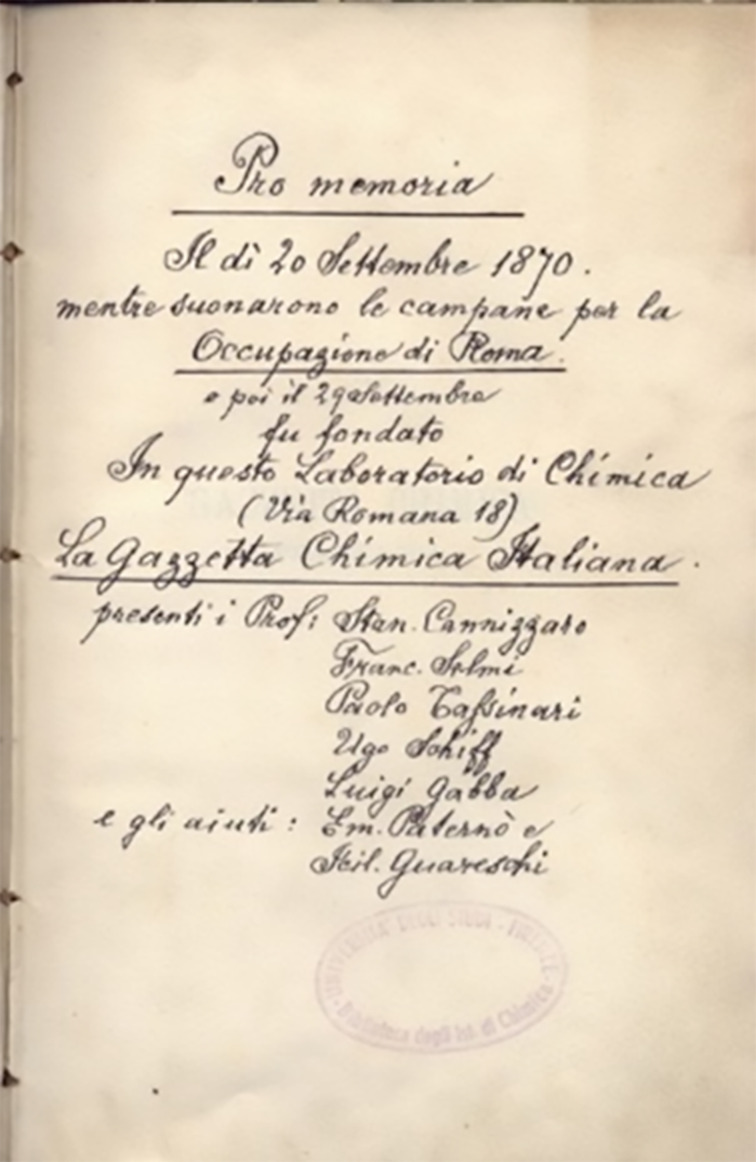
Schiff’s fictitious report on the foundation of the Gazzetta Chimica Italiana (Image reproduced from Papeo, G.; Pulici, M. “Italian Chemists’ Contributions to Named Reactions in Organic Synthesis: An Historical Perspective” *Molecules* **2013,** *18*, 10870–10900. https://doi.org/10.3390/molecules180910870, where it was published under the Creative Commons Attribution 3.0 License, http://creativecommons.org/licenses/by/3.0/.

The rugged and often nasty attitude of Schiff towards students and several colleagues was legendary; “quanto di più malvagio si possa trovare in un Tedesco quando i Tedeschi sono cattivi davvero” (the worst which can be found in a German person when Germans are really bad), according to Pietro Siccardi, who took over his position [15], and along with his parsimony has been the source of endless anecdotes (Supporting Information File 1, first paragraph). Surprisingly, Schiff developed a sincere esteem for Guareschi, who was “coming to the lab also on Sundays, working from dawn to dusk, and studying German to read the most recent literature” [16]. This esteem was evident when Schiff assigned Guareschi, at that time a 23-year-old undergraduate who had just arrived in his lab, a role in his fictitious report of the foundation of the Gazzetta Chimica Italiana, the flagship journal of the Italian Chemical Society, now part of a series of combined European journals. According to the Schiff report, the Gazzetta was established in his Florence laboratory on September 20, 1870 [17]. Florence was at that time the capital of Italy, and according to Schiff’s report, the meeting was accompanied by the joyous ring of bells celebrating the annexing of Rome to Italy and the end of the Pope secular state (Schiff held fiery anticlerical views all his life and was one of the founders of the flagship journal (L’Avanti) of the Italian Socialist Party). The two secretaries of the meeting were claimed to have been Emanuele Paternò (1847**–**1935; for a biographic sketch, see Supporting Information File 1, second paragraph) and Icilio Guareschi. This signed note by Schiff (Figure 3) is reported in many articles discussing the birth of the Gazzetta Chimica Italiana, but the official report by Luigi Gabba (1841–1916), summarized in Berichte by Adolf Lieben (1836–1914) [18] depicts a different scenario. The journal was the result of two convulsive days of discussion (September 30 and October 1) on the opportunity to establish an Italian Chemical Society. Cannizzaro strongly opposed the project, on consideration of the very limited number of active researchers existing in Italy, and also doubted that an Italian chemistry journal could achieve international recognition, something that then actually happened under the guidance of Paternò, who personally owned the journal until 1924. Icilio Guareschi was not present at the meeting. Along with Paternò, the other secretary was Domenico Amato (1839–1897), a collaborator of Cannizzaro, who had been given a technician (preparatore) position in Schiff’s group. At that time, Amato had not yet completed his education, graduating only seven years later in Naples. For unclear reasons, Amato and Schiff became archenemies, with a long legal aftermath of lawsuits between them. Unwilling to mention his archenemy in his report of the event, Schiff replaced the name of Amato with the one of Guareschi. Paternò mentions in his memories the poor esteem in which Cannizzaro held Schiff, considered unreliable as a chemist and absurdly extravagant as a person [19]. It is possible that Amato, who after leaving Florence went on to work with Cannizzaro in Rome on the structure of santonin, was responsible for this negative judgement.

**Scheme 2:**
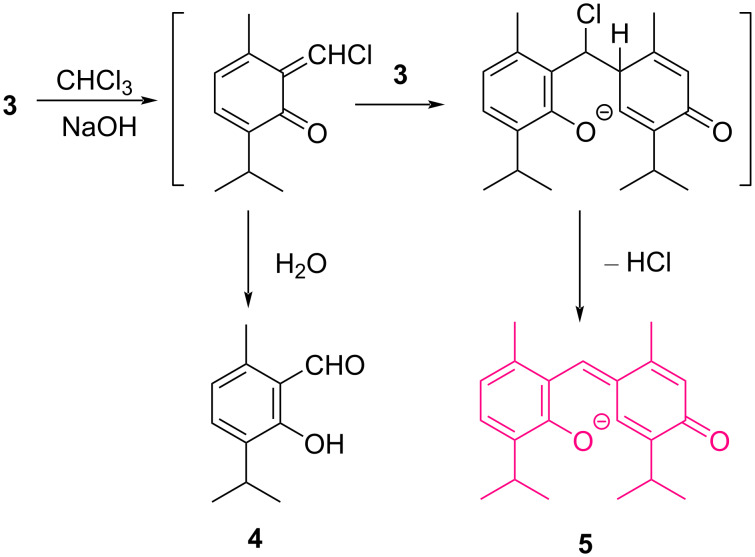
Reaction of thymol (**3**) with chloroform under the basic conditions of the Guareschi–Lustgarten reaction (right) and the Reimer–Tiemann formylation (left).

A second testimony of the high esteem of Schiff for Guareschi was the way he assigned entirely to Guareschi the discovery of the color reaction of phenols with alkali and chloroform, a reaction he reported in front of the prestigious audience of the Deutsche Chemische Gesellschaft (German Chemical Society) at the annual meeting in 1872 [20–21]. The relevance of novel color reactions for the audience can hardly be underestimated. In those times, the only methods available for the identification of compounds were holistic, that is, associated with the whole molecular construct (melting point and boiling point, optical rotation, diffraction index, density), and color reactions were therefore critical for the identification of functional groups, being the equivalent of what next became IR spectroscopy. Schiff himself had contributed to this development with the discovery of the sulfite-decolorized fuchsine test for aldehydes and with the popularization of the biuret test for peptide bonds. It is therefore natural that Schiff considered the discovery of Guareschi worth presenting at such an important meeting. What is surprising is that Schiff did not claim any role in the discovery by Guareschi that when phenols are heated with chloroform and solid KOH, a color reaction develops, with a hue dependent on the structure of the phenol. The mechanism of many color reactions popular in the 19th century is rather complex, and the one involved in the Schiff test of aldehyde is still not completely clear, despite the relevance in medical histology [22]. The report by Schiff on the Guareschi chloroform–phenol reaction attracted the attention of Karl Reimer (1845–1883) and Ferdinand Tiemann (1848–1899), who set out to investigate the chemistry involved in the color formation, eventually discovering the phenol formylation that bears their name [6]. The Guareschi reaction, then extensively applied to the identification of pharmaceutical phenols by Sigmund Lustgarten, is carried out under anhydrous conditions, either on an alkaline salt of the phenol or by adding solid KOH or NaOH to a chloroform solution of the phenol [5,21]. Reimer and Tiemann discovered that, in the presence of water, the orange color developed by phenol itself faded, and salicylaldehyde was formed [6]. The mechanism of the Guareschi reaction was clarified only in the 70s of the past century, and the relationship to the mechanism of the Reimer–Tiemann reaction became obvious (Scheme 2) [5]. Thus, dichlorocarbene generated by the basic treatment of chloroform electrophilically attacks phenol, generating a chloromethylene quinone methide. Under wet conditions, this is attacked by water, eventually leading to a formylated phenol. In the absence of water, the quinone methide is attacked by a molecule of unreacted phenate, generating the colored anion of a diphenylmethane dye that could be isolated after acidification and characterized from the reaction of thymol (**3**) and of charvachrol. The Guareschi reaction was then applied by Lustgarten to a host of medicinal phenols, and the test is included in various pharmacopoeias for the recognition of thymol (**3**) [5]. The nature of the color depends on the number of phenolic hydroxy groups and the substituents. With thymol (**3**) and charvacrol, the color is red-violet and with resorcinol blue-violet, in both cases turning to yellow upon acidification. Compared to other color reactions of phenols, the Guareschi–Lustgarten reaction is claimed to be more selective, especially for resorcinols.

While in Livorno, in 1874, Guareschi married Annetta Dassù, who died the following year after giving birth to a daughter (Maria Guareschi; for a biographic sketch, see Supporting Information File 1, third paragraph). Despite the poverty of means, Guareschi established a laboratory in Livorno, carrying out independent research in the realm of organic chemistry. In 1874, Guareschi reported the reduction of amides to aldehydes and alcohols with sodium amalgam [23], nowadays an obsolete reaction that, however, was later used by the great Emil Fischer. Two years later, Guareschi was called as a Professor of Chimica Farmaceutica (Medicinal Chemistry) at the faculty of Siena. University recruitment from high schools was not uncommon, the most famous examples in the Italian context being the ones of Alessandro Volta (1745–1827) and of the poet Giovanni Pascoli. Siena belonged to the group of Universities where teaching but not research was expected to be carried out. The Kingdom of Italy was created in 1861 by unifying not only the major Italian reigns but also a microcosms of states, the majority of which hosted academic structures. To cope with this academic redundancy, a distinction between research-based universities and teaching-oriented universities was established [24]. As a result, in 1862, Italy had more universities than France and England. In an attempt to concentrate resources, the physicist Carlo Matteucci (1811–1868), then Minister of Education, sorted the Italian universities into two classes. Class I universities (Turin, Pavia, Bologna, Pisa, Naples, Palermo, and then Padua and Rome) received funds for both tuition and research, while class II universities (all the others) were expected to carry out only teaching activities and were funded accordingly. The legal value of academic titles was independent from the university where it was obtained, but the salary of professors was 40% lower in class II universities [24]. In Siena, Guareschi started his long series of studies on naphthalene and the conversion of naphthalene to phthalides, a reaction that, two decades later, became of critical relevance for the industrial synthesis of indigo. It is surprising that, after spending only three years in Siena, Guareschi obtained, in 1879, the prestigious Chair of Medicinal and Toxicological Chemistry (Chimica Farmaceutica e Tossicologica) at the University of Turin. Guareschi was then 32 years old, and his record of publications was relatively modest, even on account of his early age. Above all, he was alien to the Piria–Cannizzaro connection that dominated academic promotions in Italy, just like Berthelot was doing in France (Supporting Information File 1, fourth paragraph). Selmi had powerful connections with the Piedmontese scientific and cultural establishment (Supporting Information File 1, fifth paragraph) and could have fostered the nomination of Guareschi in Turin, where the situation of the Chemistry Chairs was confusing. The troubles started with the (in)famous competition of 1854, where, on sheer political grounds, Raffaele Piria (1814–1865) was nominated despite the presence of two remarkable local candidates, Ascanio Sobrero (1812–1888), famous for nitroglycerin (Supporting Information File 1, sixth paragraph) and Michele Peyrone (1813–1883), famous for cisplatin [25]. Unable to decide between the two candidates, the committee opted for a third one, Raffaele Piria, who after his remarkable studies on salicin was then more interested in politics than in chemistry. After the death of Piria in 1864, the Chair eventually was assigned to Adolf Lieben (1836–1914), the discoverer of the iodoform test. Lieben came from the Paternò group in Palermo, from where he had been forced to move by the Ministry of Education itself, who considered it a waste of resources to have two prestigious chemists (Lieben and Cannizzaro) at the same university. After only four years, Lieben moved to Prague, and in 1871, his Chair was offered to Paternò, who declined the nomination. Schiff held the Chair from 1876 to 1879. Unhappy with the situation of the laboratories, Schiff, whose motto was “I love scholars, not students” (amo gli studiosi, non gli studenti) [15], annoyed the students to the point that one evening, he was ambushed, his head wrapped in a sack, and clubbed by the angry students (after this aggression, his pleas to return to Florence were eventually satisfied). The establishment of the Faculty of Pharmacy in 1874 further aggravated the situation of chemistry in Turin, since an additional Chemistry Chair became vacant. This was the academic setting when Guareschi moved to Turin in 1879.

## The first two decades of the Turin years (1879–1900)

**Figure 4:**
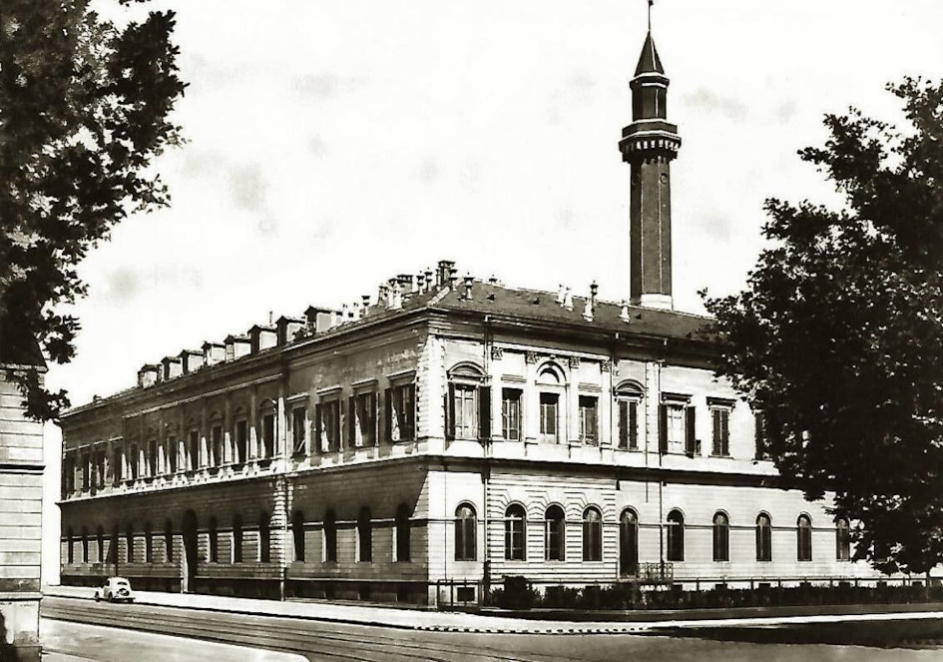
The chemistry building of Turin University in a historical picture. Note, that one of the “mysterious minarets” that Primo Levi wrote in his work The Periodic Table. Levi tells that the chemistry students rumored that it “had been built at his bidding (Giacomo Ponzio, the Head of Department) in his remote youth, in order to celebrate there each year a foul and secret orgy of salvage. During it all the past year’s rags and filter papers were burnt, and he personally analyzed the ashes with beggarly patience to extract from them all the valuable elements (and perhaps even less valuable) in a kind of ritual palingenesis with only Caselli, his faithful technician beadle, was authorized to attend.” (Levi, P. *The Periodic Table*; Schocken Book: New York, NY, 1984; p 33. Source: photographic fund of the Sistema Museale di Ateneo, Archivio Scientifico e Tecnologico dell'Università di Torino). Reused with permission by ASTUT (Archivio Scientifico e Tecnologico dell’Università di Torino). This content is not subject to CC-BY 4.0.

In the twilight of the 19th century, Turin was a cradle of culture, with a remarkable capacity to attract and foster talents. Walking in the elegant center of Turin, one could have met the (nowadays highly controversial) criminal anthropologist Cesare Lombroso (1835–1909), the mathematician Giuseppe Peano (1858–1932), Giulio Bizzozzero (1846–1901, the discoverer of the role of platelets in blood coagulation and of the hematopoietic activity of bone marrow), Edoardo Perroncito (1847–1906, the founder of occupational medicine), Galileo Ferraris (1847–1897), famous for the rotating magnetic field, or even the philosopher Friedrich Nietzsche (1844–1900), who considered the city “a paradise for the eye and the feet (Nietzsche loved taking long walks), a place that one does not want to leave” and wrote there, inter alia, Ecce Homo and his anti-Wagner pamphlets [26]. When Guareschi arrived in Turin, the chemistry laboratories were still located at the Monastery of Saint Francis, a huge downtown building secularized during the Napoleonic period, when Piedmont had been annexed by France. Part of the building was bombed during WWII, but the aisle occupied by the chemistry lecture room still exists. The building nowadays hosts the Medical Academy and is of great relevance to chemistry, being where, downtown the capital of the Kingdom of Sardinia (sic!), Ascanio Sobrero synthesized nitroglycerin in 1849 [24] and where Bartolomeo Gosio (1863–1944) reported the purification of the first antibiotic (mycophenolic acid) in 1899 [27]. Guareschi spent his first 15 years in Turin in a poorly equipped laboratory, where he was working in what he describes as a “communist society” since students, assistants, and professors were sharing the same space. With Guareschi as the Chair of Pharmaceutical Chemistry and the call of Michele Fileti (1851–1914) to the Chair of Chemistry, Turin finally reacquired the international visibility in chemistry it had enjoyed with Sobrero and Peyrone and that the fatal contest of 1854 had eclipsed for more than a quarter of a century. In 1883, Fileti reported the first synthesis of indole and scatole, soon dwarfed in terms of relevance in the following year by the discovery of the more general Fischer synthesis. In 1894, a novel chemistry building was inaugurated in front of the Valentino Park (Figure 4), a then peripheral area of Turin, where it is still currently located. It was the largest chemistry building in Italy, hosting the faculties of Chemistry and of Pharmacy and is immortalized in some chapters of The Periodic Table by Primo Levi, who graduated there in 1941. Guareschi moved to the new institute before Fileti, who, paradoxically, stopped doing active research in those years, at the early age of 47.

In Turin, Guareschi pursued an amazing range of research topics. The one of ptomaines (alcaloidi cadaverici, cadaverous alkaloids) and the chemistry of naphthalene led to considerable fame for Guareschi because of both his original contributions and his comprehensive reviews of the area. Thus, his Introduzione allo Studio degli Alcaloidi con special riguardo agli alcaloidi vegetali ed alle ptomaine (An Introduction to Alkaloid Chemistry, and especially on those of plant origin and on ptomaines), first published in 1892, was translated four years later into German, and the popularity of this work is testified by Willstätter, who in his memoirs refers to Guareschi as “alkaloid chemist” [28]. Ptomaines (from the Greek πτωμα, cadaver) are anaerobic bacterial degradation products of proteins and choline-containing phospholipids. The formation of these is associated with putrefaction of animal and plant tissues. Many ptomaines are polyamines (putrescine, cadaverine), but others are not (indole, scatole). The name ptomaine (Leichengift in German) has disappeared from the modern lingo of organic chemistry because of the heterogeneous nature of these compounds. The formation of ptomaines was considered a major cause of food poisoning, a huge problem throughout the 19th century that, as we now know, is rather associated with bacterial activity. The surprising popularity of ptomaines as research topic was also due to the forensic relevance. Thus, ptomaines yield positive test results for alkaloids and can interfere with the color reaction and tests used to detect plant poisons in forensic investigations. There is currently a resurrection of interest in nitrogenous small molecules associated with corpse decomposition since the profile of these compounds can be useful to assess postmortem intervals and to locate human remains in wild environments [29]. Additionally, the human ptomaine profile is significantly different from the one of other mammals and can differently affect soil as well as vegetation growth and foliage color [30]. From a historical standpoint, the name ptomaine was coined by Francesco Selmi (1817–1881), who died in 1881 because of an infection he had contracted while dissecting the corpse of an animal affected by typhoid fever. The chemical characterization of ptomaines was carried out by German (Nencki, Brieger) and French (Gautier) chemists, and Guareschi’s contributions to the area are in the realm of analytical chemistry and in the systematic organization of the topic. In the course of these studies, Guareschi also isolated succinic acid from meat, the first report of this Krebs cycle intermediate from muscle tissues. The monograph on ptomaines [31] that Guareschi wrote in 1883 with the physiologist Angelo Mosso (1846–1910) for a biographic sketch (see Supporting Information File 1, seventh paragraph) became popular all over Europe and is full of surprising observations, such as the one that frogs poisoned with a ptomaine mixture isolated from the putrefaction of blood fibrin systematically emit a pleasant odor similar to the one of orange flowers (sic!). In 1885, Guareschi, Cannizzaro, and Paternò prepared a report for the Italian Government on the relevance of ptomaines in forensic analyses. In the same years, Guareschi published various articles on the chemistry of naphthalene. The last one dates from 1887 and deals with the formation of isomers in the electrophilic aromatic substitution of naphthalene with chlorine and bromine [32]. This work was done in collaboration with Pietro Biginelli (1860–1937), the only collaborator of Guareschi who achieved fame in the realm of organic chemistry [33].

**Scheme 3:**
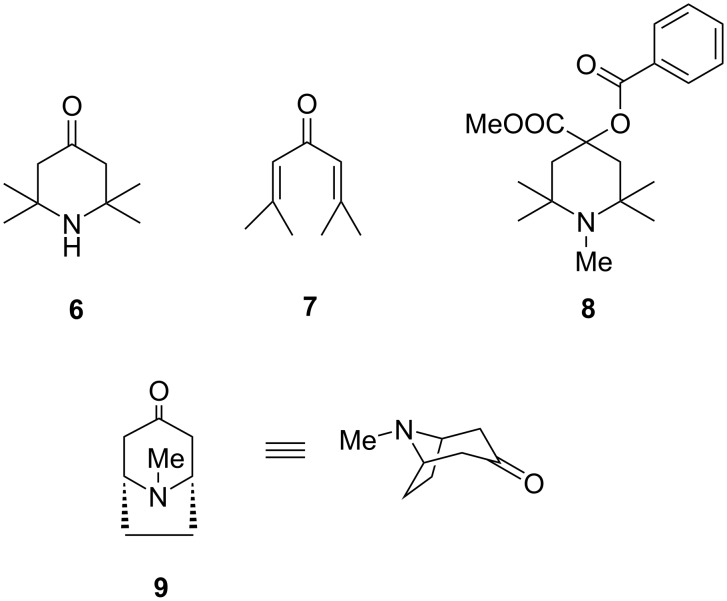
Triacetonamine (**6**) and the related compounds phorone (**7**), α-eucaine (**8**), and tropinone (**9**).

The availability of novel and spacious laboratories is associated with the most important contributions of Guareschi to organic chemistry, those on piperidines and pyridines. His first article in the area appeared already in 1891 [34] and the last one posthumously in 1919 [35]. In 1894, Guareschi reported the first high-yield synthesis and the structure elucidation of triacetonamine (**6**, Scheme 3) [35–37], the sterically hindered amine that is the starting material for the synthesis of the popular oxidant TEMPO [38]. Triacetonamine had first been reported in 1874 and was synthesized by refluxing of a saturated solution of ammonia in acetone, but the structure of this trimer had not yet been established [39]. Guareschi obtained triacetonamine in high yield (>70%) by treatment of phorone (**7**), the trimer of acetone, with ammonia and established the correct structure, ending two decades of controversies. Triacetonamine is an important chemical feedstock, used for drug synthesis and the production of flavors, and nowadays also has electronic applications. The Guareschi synthesis of triacetonamine was industrialized by Schering in connection with the introduction of α-eucaine (**8**) in medicine, the first synthetic local anesthetic, a compound devoid of narcotic properties and with an improved hydrolytical stability compared to cocaine [40]. Eucaines were used as anesthetics during WWI. Before the war, England used to import them from Germany, and to replace the import, Jocelyin Thorpe (famous for, inter alia, the Guareschi–Thorpe reaction) was involved in the production at Imperial College [41]. Guareschi had not patented his synthesis of triacetonamine, making Willstätter comment that Schering “should at least have sent one kilogram of triacetonamine to Guareschi” [42]. Triacetonamine bears a certain similarity to tropinone (**9**), the heterocyclic core of tropane alkaloids, and the one-pot preparation from acetone and ammonia reminds of, and anticipates by two decades, the Robinson synthesis of tropinone and the Willstätter synthesis of the corresponding 2-ethoxycarbonyl derivative [43], two reactions dating from 1917 and also based on iterative intramolecular Mannich reactions.

Guareschi supported a distinction between scientific and industrial developments and claimed that, unlike many colleagues from the rest of Europe, who had accumulated great fortunes thanks to their studies (Gay-Lussac, Dumas, Liebig, and Hofmann are the names that he cites), Italian scientists had pursued the study of nature only for the sheer love of research, dismissing the financial benefits that could have come from the industrial applications of their discoveries [44]. Guareschi’s view echoes the inscription that the Jesuit Lazzaro Spallanzani (1729–1799) posed in Pavia’s laboratory where he accomplished the first in vitro fecundation: “Quid hic? Intueri Naturam. Quo munere? Curiosum esse” (What is done here? We study Nature. For what reason? Because we are curious) as well as the answer given by Röntgen when he was asked why he had not patented X-rays (“They were invented by Nature, not by me.”) [24]. Rather than a moral virtue, this lack of connection with industry is nowadays identified by historians as a major impediment to a robust development of science in Italy during the 19th century [24]. In many academic settings, patenting was not deemed suitable for university professors. When Galileo Ferraris (1847–1897) was asked why he had not patented his discovery of the induction motor, he answered, “I am a professor, not an industrialist” (incidentally, Tesla obtained his patent on this motor in 1888, 43 days after the publication of the details of the 1885 discovery by Ferraris on the generation of a rotating magnetic field. The patent of the Serbian inventor was, however, apparently based on previous independent studies, according to the most recent studies on the endless question of the Ferraris–Tesla priority) [24]. Similar reasons underlie the lack of interest of Sobrero, who was professor of applied chemistry (sic!), to patent his discovery of nitroglycerin, rather recommending “never to use it” because of the devastating explosive power [25].

**Scheme 4:**
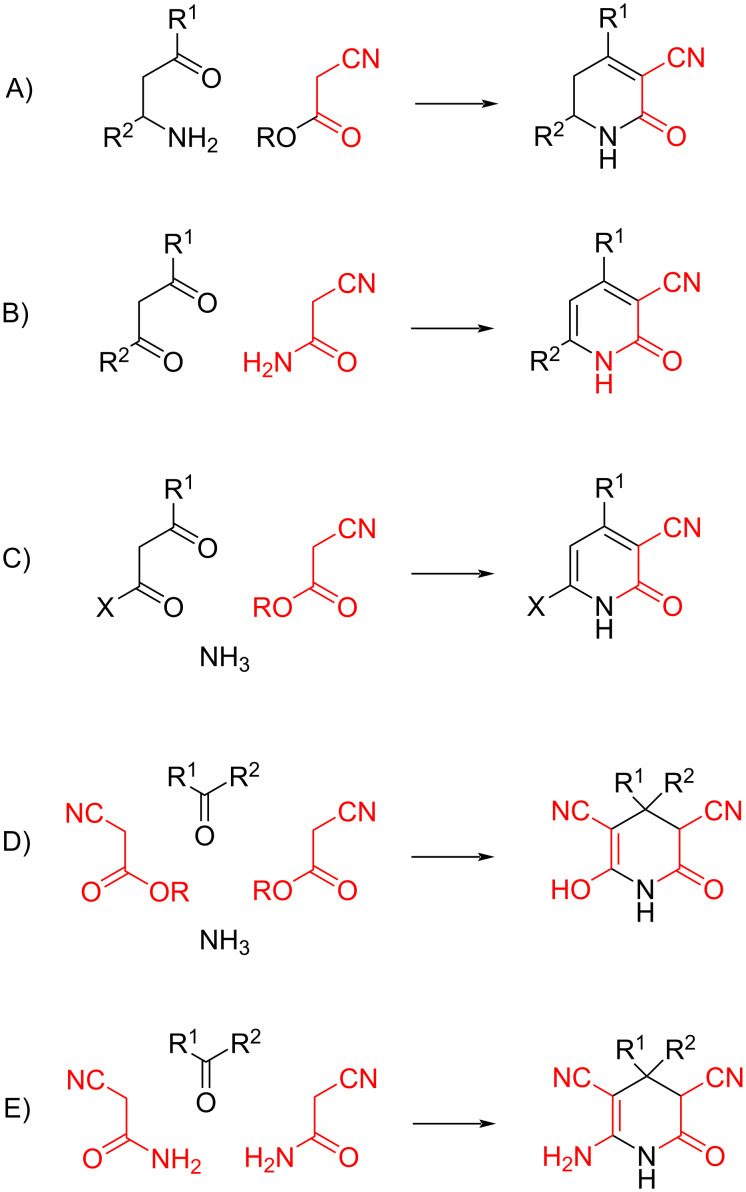
Taxonomy of the Guareschi pyridone syntheses.

Triacetonamine aside, the contributions of Guareschi to the chemistry of heterocycles are classically declined in his cyanopyridone syntheses, a topic on which Guareschi published 16 notes, most of them in the last decade of the century. Research in this area was possibly inspired by his own work on ptomaines and by the one of Fileti on cyanamide. At least five different reactions are pooled under the name “Guareschi or Guareschi–Thorpe synthesis of pyridines”, and considerable confusion exists in the literature that apparently makes no distinction between the various reactions (Scheme 4). Part of the confusion is related to the publications of the original work by Guareschi in a “local” scientific journal (Atti della Reale Accademia delle Scienze di Torino). The journal was well known, but since Guareschi’s articles were written in Italian, they were basically disseminated via the Chemisches Zentralblatt summary. Additionally, ambiguities exist on the molecularity of the various reactions since Guareschi was often preparing β-aminocarbonyl derivatives in situ from the self-condensation of acetone and the aza-Michael trapping of mesityl oxide. This method works well only for acetone, while further work used preformed β-aminocarbonyl compounds. The classification we propose is based on the molecularity of the reaction (two, three, or four components), calculated based on preformed β-aminocarbonyl species and the use of cyanoacetic esters or cyanoacetamide as the cyanocarbonyl reagent. We suggest to name type-I Guareschi pyridone synthesis the two-component reaction between a β-aminocarbonyl and a cyanoacetic ester, a reaction first reported in 1893 [34]. It is a classic combination of a Knoevenagel condensation and ester aminolysis typical for many heterocyclic syntheses. The type-II Guareschi pyridine synthesis is a modified two-component version, mechanistically similar to the Biginelli pyrimidine synthesis [33] and based on the condensation of cyanoacetamide and a β-dicarbonyl derivative [45–46]. This is the most famous Guareschi pyridine synthesis, both in textbooks and in other literature. To avoid the formation of two isomers, the reaction is generally carried out with β-ketoesters, α,γ-dicarbonyl esters, β-ketoaldehydes, or in later modifications acetylenic ketones [47–48]. The reaction was used for the 1939 commercial synthesis of pyridoxine (vitamin B_6_) [2], the first industrialized total synthesis of a nontrivial natural product. The original experimental protocol was recently improved by replacing the inorganic base (KOH, NaOH) with the amidine base diazobicycloundecane (DBU) as well as ethanol with *n*-propanol [49]. The type-III Guareschi reaction is a three-component reaction reported in 1898 [50]. It involves the reaction of a β-dicarbonyl (ester or diketone) with a primary amine or ammonia and a cyanoacetic ester. The mechanism of this condensation closely resembles the three-component version of the Hantzsch pyridine synthesis and generates 6-hydroxypyridones. Also mechanistically similar to the Hantzsch pyridine synthesis is the Guareschi type-IV pyridine synthesis, first reported in 1897 [7] and involving the four-component condensation of ammonia, a carbonyl derivative, and two molecules of a cyanoacetic ester. The reaction affords 3,5-dicyanoglutarimides, known as Guareschi imides, which, under the original protocol, is limited to ketones as the carbonyl component. When aldehydes are used, the adduct is aromatized to the corresponding 2-hydroxypyridone [50–51]. Finally, a modification of the type-IV Guareschi reaction that involves the condensation of two moles of cyanoacetamide (and not a cyanoacetic ester) with a carbonyl derivative (aldehyde or ketone) was further investigated by the British chemist Jocelyn Fred Thorpe (1872–1940), a decade after the original studies by Guareschi [52]. In Thorpe’s modification, the reaction takes place in the presence of a secondary amine, affording a 6-aminopyridone, which, after acidic treatment, is turned into the same β,β-disubstituted glutarate formed by hydrolysis of the pyridone from the type-IV Guareschi reaction. This modification of the type-IV Guareschi reaction is the “real” Guareschi–Thorpe reaction, which in many cases gives a better yield of the glutarates compared to the original protocol. However, this reaction was confused with the other Guareschi syntheses that ended up being referred to in the literature as Guareschi–Thorpe reactions. Thorpe himself acknowledged that the compounds formed in his modification of the type-IV Guareschi reaction are “the same as those produced by the well-known reaction discovered by Guareschi” and refers to the Guareschi reaction in the title of his publication [52].

The type-IV Guareschi and the Guareschi–Thorpe reactions are the most straightforward entry into β,β-disubstituted glutarates, and the type-IV Guareschi synthesis is used in the preparation of the conformationally constrained γ-aminobutyric acid (GABA) analogue gabapentin (**2**) [3], a blockbuster drug for the management of neuropathic pain also used for a range of off-label indications that includes hot flashes and restless leg syndrome [53].

**Scheme 5:**
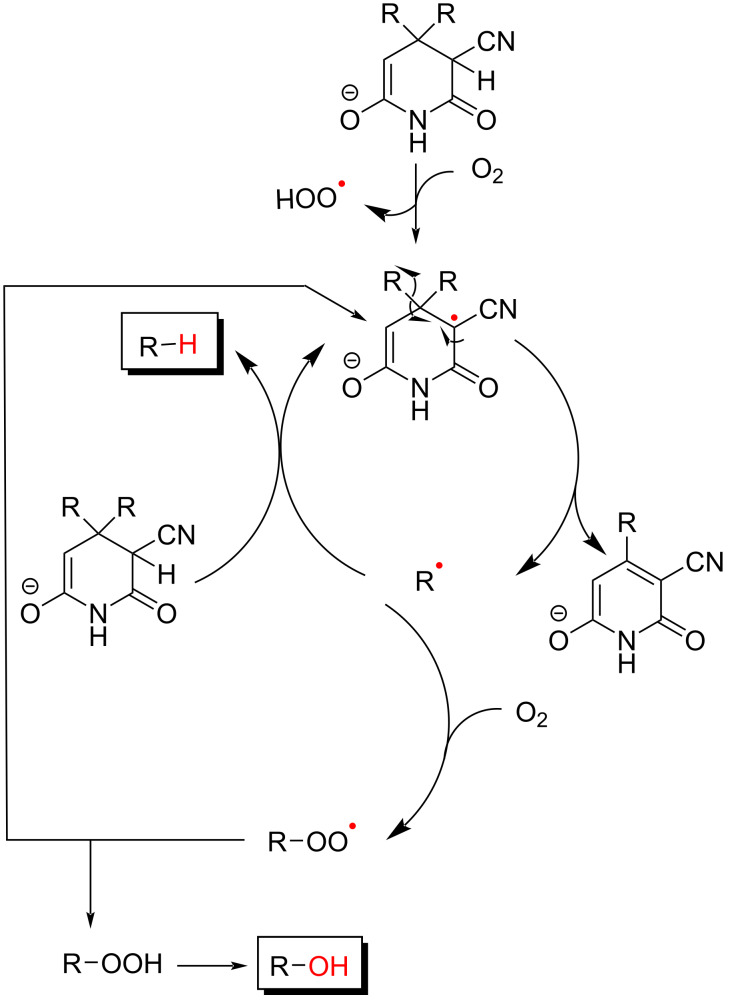
The catalytic cycle of the “1897 reaction”.

Guareschi imides exist in solution predominantly as dicarbonyl tautomers with a *cis* arrangement of the cyano groups. Conversely, the corresponding anions exist as a mixture of mono- and dienolized tautomers, with a strong preference (≈9:1) for the monoenolized form [8]. Guareschi imides have been extensively investigated because of their bioactivity profile, the use as building blocks to access other classes of heterocycles as well as quaternary centres, and their remarkable chemistry [54]. By far, the most intriguing reaction of these is the so-called “1897 reaction”, formally an internal redox reaction where the heterocyclic system is oxidatively aromatized and one of the substituents at C-4 reductively lost as a hydrocarbon. The reaction involves the treatment of a ketone with a solution of ethylcyanoacetate in ethanolic ammonia [7]. A precipitate of the ammonium salt of the imide slowly forms. After stirring for 24–36 h, the precipitate is filtered, and the filtrate, after washing with ethanol or diethyl ether, is acidified to precipitate the imide. In the course of the work-up, Guareschi noticed the formation of copious gas, bubbling from the solution of the ammonium salt of the dicyanoimide. A similar bubbling could be observed when the crystallized imide was treated with ammonia. Guareschi collected the gas evolved from the reaction, and much to his surprise, identified it as the hydrocarbon corresponding to the more substituted alkyl residue of the starting carbonyl derivative. Guareschi found that the fragmentation was quantitative when an ethanolic solution of the imide was treated with magnesium hydroxide, poorly soluble but sufficient to ionize the cyanopyridone. It should be pointed out that the identification of gaseous compounds was not at all trivial before the introduction of GC–MS, and the elemental analysis of gases required special attention [55]. Guareschi also made the observation that air was necessary for the development of the gas, proposing a general scheme for the transformation that provides, however, no clue for a possible mechanism. In terms of overall redox transformation, the reaction formally “hydrolyses” a ketone into a hydrocarbon and a carboxylic acid, and the mechanism remained a black box for over a century, being eventually clarified only in 2007 by a team of British and Russian chemists [8]. One important starting observation was that the presence of radical traps, such as 4-hydroxy-substituted TEMPO, was detrimental for the reaction, which evidently involved free radicals. Anaerobic conditions (degassing with argon) inhibited the reaction, which could be restarted when oxygen was bubbled into the solution. This observation showed that oxygen, although not involved in the stoichiometry of the reaction, was critical. The alcohol corresponding to the alkyl residue was detected as a side product and became the main reaction product with substrates that generated stabilized radicals such as benzyl radicals. The formation of alcohol byproducts confirmed the homolytic nature of the bond cleavage at C-4 and served to assess the half-life of the alkyl radical generated. To this purpose, the reaction of dicyanoglutarimides bearing a radical clock substituent (cyclopropylmethyl or 5-hexenyl) was investigated. The reaction of the first substrate gave a mixture of but-1-ene and but-3-en-1-ol, in accordance with the loss of the C-4 alkyl group as a radical, since the formation of a cylopropylmethyl cation would have generated a mixture of cyclobutanol and cyclopropylmethanol. The second substrate gave a mixture of cyclopentylmethanol and 5-hexenol. Since the half-life for the rearrangement of the 5-hexenyl and the cyclopropylmethyl radical are known, it could be concluded that under the reaction conditions, the half-life of the radical lost from C-4 was >1 μs. When the reaction was carried out in ^17^O-enriched water, the alcohol side product was not labeled, suggesting that alcohols are formed by radical trapping by oxygen and the intermediate formation of hydroperoxides. Conversely, cations would have been trapped by water, resulting in the formation of labeled alcohols. Cyclic voltammetry experiments showed an irreversible one-electron oxidation at 0.80 V, in accordance with the involvement of oxygen in the reaction. Remarkably, when the glutarimide was labeled with deuterium in position 3, the deuterium labeling was transferred to the hydrocarbon liberated in the reaction, showing that hydrogen abstraction was carried out by the alkyl residue lost from C-4. Based on these observations, a mechanism was proposed, triggered by hydrogen abstraction from C-3 by oxygen (Scheme 5). The stabilized radical generated in this way acts then as a chain carrier, aromatizing by loss of one of the alkyl residues at C-4. In turn, this next abstracts hydrogen at C-3, regenerating the carrier and continuing the cycle. The C–H bond at position 3 of Guareschi imides is unusually weak. Hydrogen abstraction by an alkyl radical and formation of a hydrocarbon C–H bond is strongly exothermic (calculated enthalpy change: ≈20 kcal⋅mol^−1^) and favored over abstraction by a hydroperoxy radical, which would be only exothermic by 2 kcal⋅mol^−1^. The exothermicity of the hydrogen abstraction reaction is reduced with stabilized radicals, which have weaker C–H bonding. In this case, trapping by dissolved oxygen competes, generating a hydroperoxy radical that can next evolve into an aldehyde by β-elimination of water, or after reduction, into an alcohol.

**Scheme 6:**
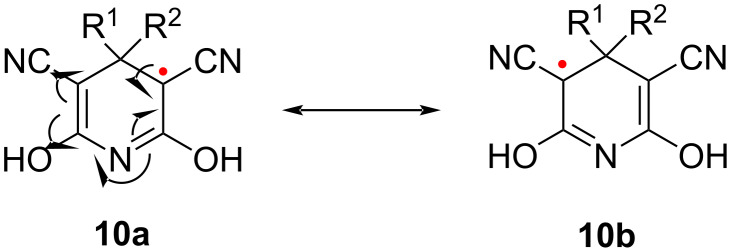
Resonance forms of the radical **10**.

The stability of the radical **10** is surprising. Stable radicals (O_2_, NO, nitroxyl derivatives, DPPH) are characterized by a two-center three-electron bond [56]. Conversely, the stability of **10** is entirely due to mesomeric effects, as expressed by the possibility to formulate two identical resonance forms, **10a** and **10b**, for the undissociated system (Scheme 6).

It has been remarked that life is basically a hydrogen transfer to carbon dioxide, outrageously approximated by the equation CO_2_ + 2H_2_ → CH_2_O + H_2_O [57]. The energy to fuel the process is associated with the vectorial chemistry of the electron transfer chain and the mitochondrial chemiosmotic generation of ATP, a suggestion by Mitchell long considered heretic in the biochemistry community, which searched for decades the highly reactive intermediate capable of transferring a phosphate group to ATP. The Guareschi “1897 reaction” shows a basically different strategy to generate reductive power, associated to aromatization of a prearomatic substrate. The chemiosmotic generation of energy is as universal as the genetic code, and the origin of this is considered the central puzzle of biology [58]. Hydrogen cyanide is a fundamental prebiotic constituent, and glutarimides are easily formed under prebiotic conditions [58]. Could a Guareschi amide precursor have served as a primordial source of reducing power in prebiotic media en route to the development of the chemiosmotic way to energize life [58]? The “1897 reaction” is also at odds with the basic tenet in geosciences that the generation of hydrocarbons is an anoxygenic process [59]. Anticipating the rationale of the Fischer–Tropsch process, Guareschi suggested that his reaction could be used to produce oil, a finite resource, or even be involved in the generation of oil. Guareschi maintained an active interest in the origin of oil, with his first publication being the study of a fossil resin, and wrote two monumental monographs on this topic [42]. Despite intense geological studies, some disagreement still exists today on the origin of oil, and the organic theory that views oil as the result of thermal alteration of organic material in sedimentary rocks is not universally accepted. In his patriotically-biased view of science, Guareschi ascribed the abiogenic theory of oil formation to Spallanzani and the organic theory to Volta, the discoverer of methane. In the second half of the 19th century, the abiogenic theory was supported not only by its paladin von Humboldt, but based on laboratory experiments, also by eminent chemists such as Mendeleev (1834–1907) and Berthelot. Their ideas had a strong influence on Guareschi, who, after first supporting the organic origin of oil, later became a moderate supporter of the abiogenic theory.

The first publication on pyridines appeared while Guareschi was actively contributing to the first edition of the Italian Pharmacopoeia (Farmacopea Ufficiale del Regno d’Italia), which appeared only in 1892, three decades after the unification of Italy. Guareschi next produced a more than 1000-pages-long commentary on the Pharmacopoeia, where all the entries are discussed in detail, presumably using the material used to elaborate the Pharmacopoeia [60].

## The last two decades of the Turin years (1900–1918)

**Figure 5:**
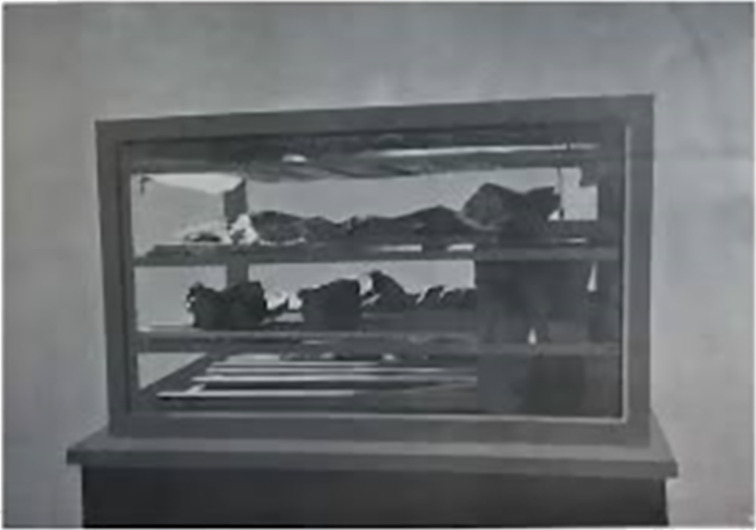
The wet chamber used by Guareschi to restore parchments (Gorrini, G. L'incendio della R. Biblioteca Nazionale di Torino, Torino-Genova, Renzo Streglio e C. Editori, 1905, p. 187).

The first decade of the past century was a magic moment for science in general and for the Italian organic chemistry community in particular, with Ciamician and Paternò setting the foundation of organic photochemistry in what has been cogently named “the Bell’Epoque of photochemistry” [61]. Guareschi was, however, alien to the exciting developments of those years. The research on pyridines and, in general, his organic chemistry studies were gradually phased out due to a growing involvement in issues of national relevance that, in turn, moved his interests first to technology and history and next to nutrition and war gases. The first event was the fire at the Turin Biblioteca Nazionale Universitaria (BNU, National University Library) in 1904 and the second one was WWI. The Turin BNU was one of the major Italian libraries, hosting 30,000 ancient volumes, 4,500 manuscripts, and over 1,000 incunabula. It is nowadays known for the Fondo Foà-Giordano, a later acquisition that represents the major source of Vivaldi manuscripts [62]. On the night of January 25, 1904, a fire destroyed more than two thirds of the volumes and half of the manuscripts hosted in the library, including the only copy of Très Belles Heures de Notre-Dame by Jean de Berry, illustrated by Jan van Eyck. It was a veritable “European cultural tragedy”, as commented by contemporaries. The books and manuscripts on paper recovered from the fire could be restored, but the state of those on parchment was dramatic since the combined action of the heat from the fire and the water used to extinguish it had reduced them to compact blocks of shrunken, coalesced, and partially carbonized pages. The Minister of Education, Vittorio Emanuele Orlando, gave Guareschi the task of devising a method to recover them. To this purpose, Guareschi perfectioned a device known as “wet chamber” (camera umida), where the shrunken coalesced pages could be hydrated without becoming wet (Figure 5). In this way, the ink and paint presented on the collaged matrix could be preserved. Each parchment sheet was then sterilized with phenol and formaldehyde to avoid microbial degradation and eventually flattened to partially recover the original size. Thanks to this very labor-intensive work, almost 3,000 parchment pages could be recovered [63]. Guareschi is considered the father of the scientific approach to restauration and the initiator of studies on the preservation of parchments, an area which received enormous attention after the discovery of the Dead Sea scrolls in the 40s of the past century. The physical and chemical bases of the process used by Guareschi were clarified only recently by differential scanning microscopy studies [64].

In connection with the work on the restauration of parchments, Guareschi developed an interest in the chemistry and technology of ancient inks and dyes, a topic he covered in some monumental monographs [65]. These, and the study on the history of chemistry, filled most of his scientific life in the first decade of the century. In particular, Guareschi analyzed and commented Plichto by Giovanventura Rosetti, a major Renaissance treatise of painting, the first edition (1542) of which Guareschi located at the British Museum in London and compared to later editions present in Italian museums. The almost maniacal interest of Guareschi for Plichto and the technology of ancient inks seems paradox when compared to the developments in chemistry in those years. Guareschi also started to publish extensively on the history of chemistry, in particular on French chemists (Lavoisier, Berthelot), also writing a biography on Selmi [64]. In 1911, he headed the committee of the Turin Academy of Sciences for the publication of a selection of Avogadro’s works [66]. Avogadro died in 1856 and remained little known to the scientific community until Cannizzaro popularized his theories at the Karlsruhe conference of 1860. Therefore, the publication of selected works played a critical role to firmly establish the role of Avogadro in modern science. Guareschi’s monograph on Avogadro was translated into German and included by Ostwald in the book series of classics in science [65]. The involvement of Guareschi in the first edition of the Italian Pharmacopoeia was accompanied by the production of an extensive commentary to be used by both pharmacists and industrialists. In connection with his interest in chemistry and art, Guareschi described a color reaction for the selective detection of bromine in the presence of other halogens [67]. The test is based on the action of sulfitic leucobases of triphenylmethane dyes, such the decolorized fuchsine (Schiff reagent), which develops a deep violet color in the presence of traces of bromine. The reaction is very sensitive and selective for bromine in the presence of other halogens. Using this test, Guareschi could demonstrate the presence of traces of bromide or brominated compounds in a variety of matrixes, including food and tissues (Supporting Information File 1, eighth paragraph). The paternity of this reaction was contested by the French chemist Georges Denigès (1859–1951), who, however, published his discovery of the same reaction six months after the disclosure by Guareschi. The ability of the test to distinguish between iodine and bromine is remarkable and difficult to explain in the light of the mechanism by which the Schiff reagent reacts with aldehydes and eventually regenerates a triphenylmethane dye from the sulfite adduct [22]. The study of old color reactions has not only mechanistic interest but can lead to new drug candidates, as shown by the cannabinoquinoid VCE-004.8, a compound inspired to a hashish color reaction and currently under Phase II human clinical study for scleroderma, an autoimmune disease [68].

**Figure 6:**
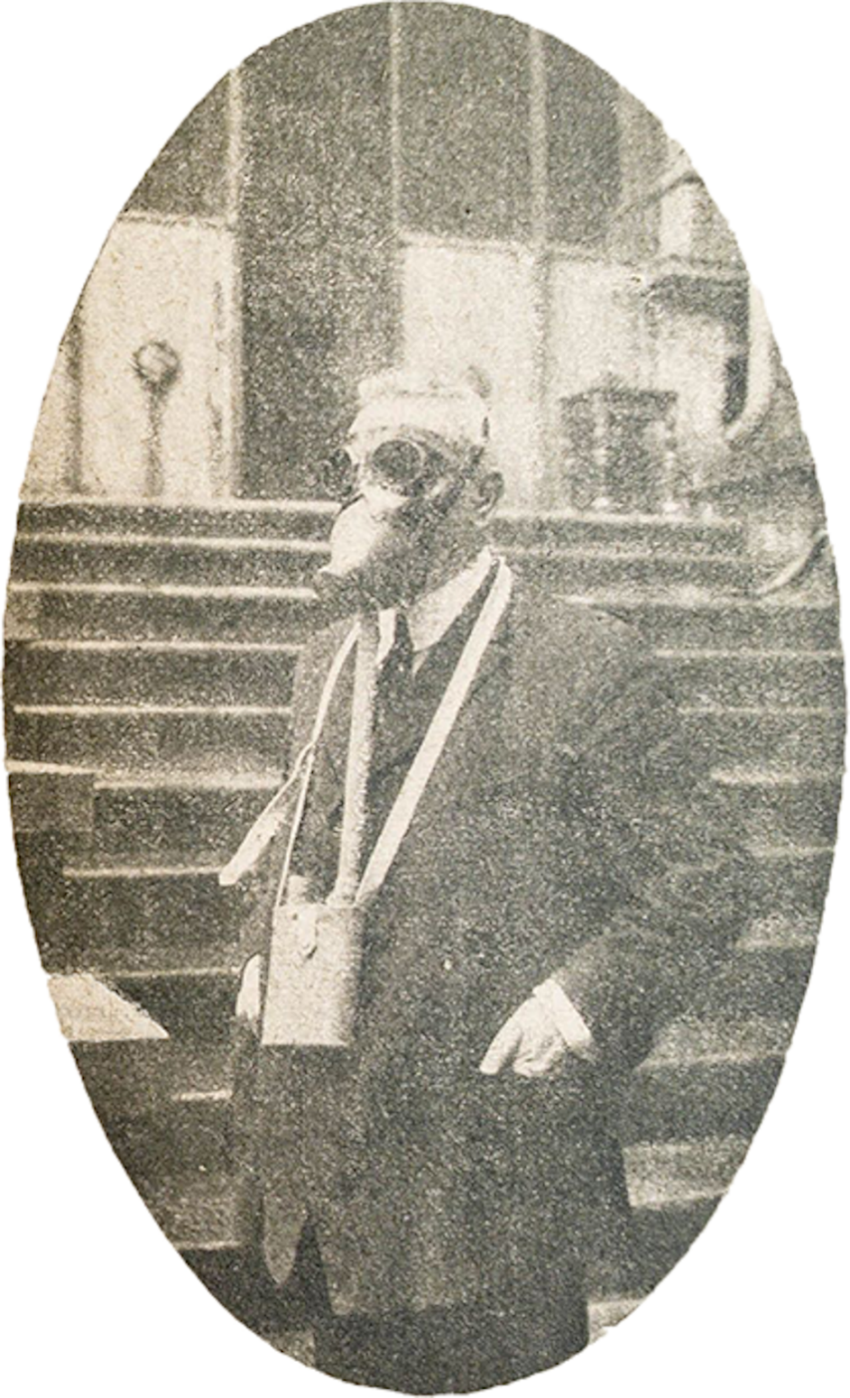
The Guareschi mask. (Servizio Chimico Militare. L*'opera di Icilio Guareschi precursore della maschera a filtro contro i gas asfissianti*. Esposizione Nazionale di Chimica, 1925, Torino (Stadium) p. 11). This content is not subject to CC-BY 4.0.

This relatively quiet life of historical and analytical activities had an abrupt end with the beginning of WWI, which shifted the interest of Guareschi to the needs imposed by the war. When war broke out in the summer of 1914, the Italian scientists split. The mathematicians, by far the largest academic community (40% of the Italian academic chairs as of 1900) [69], backed up war against Austria, sometimes even fanatically (Supporting Information File 1, ninth paragraph [9]). Conversely, the chemical community, aware of the industrial inferiority of Italy compared to the other belligerents, supported neutrality or even war against France and England, leading to the accusation of having been seized by German science. These allegations were especially aimed at Ciamician, who was born in Trieste, then part of the Austro–Hungarian Empire, and had graduated in Germany (at Giessen, the only German University accepting students who had not followed classic studies). He had German collaborators (Silber, Dumstadt [70]) and was even Germanizing the Italian name of his collaborator Sernaggiotto into von Sernaggiotto in his articles in Berichte [19]. Paternò, vice president of the Senate, prepared a letter to the King claiming that, after three decades of alliance with the Central Empires, “pacta servanda sunt”, and that Italy should militarily support Germany and Austria in the war [71]. Ciamician and Guareschi backed neutrality on the ground that the war was a crime against reason and everything the European civilization had accomplished [69]. Shaken by the events, Guareschi started to write a diary where he commented the political and military development of the war. It is an interesting read and a vivid document of how the widespread perceived "superiority of the European civilization" permeated even the most advanced sections of society. Thus, Guareschi, echoing the Aufruf an die Kulturwelt (Fulda declaration) he had received from Willstätter, was outraged by the deployment of colonial soldiers (African, Mongolian, Indian, Canadian (presumably indigenous Americans)) by the Triple Entente. However, when Italy entered the war in May 1915, objection of conscience was not an option for many, and personal opinion and professional activities could often not be separated anymore*.* “Le patriotisme, c'est aimer son pays. Le nationalisme, c'est détester celui des autres.” is a histrocial quote by De Gaulle that may aid to reenact some of the views at that time. As the carnage was going on and his students were dying at the front, Guareschi shifted his interest first to the development of a gas mask and next to the issue of famine that was looming on the Italian population. In the wake of Mosso’s studies on Alpine physiology, Turin became a hot bed of research on war. The efficiency of the swab-type Ciamician–Pesci gas mask adopted by the Italian army was questioned from the very beginning. Annoyed by the activities of colleagues who “pretend to be chemists”, Guareschi chaired the committee of the Industrial Association of Turin for the defense against war gases. Guareschi was a professor of toxicology and had considerable expertise in the analysis of gases, having published important contributions on the elemental analysis of gases. With the assistance of his son Pietro, a civil engineer, he developed a mask based on soda lime, a material that, as a fine powder, he considered capable of neutralizing all chemically reactive gases used in chemical warfare [72]. It is surprising how Guareschi identified, within a few months, soda lime as the ideal neutralizing agent for war gases, something that took all belligerents years to realize. We can surmise that his experience with the elemental analysis and the separation of gas mixtures, coupled with his work on ptomaines, had made him aware of the remarkable effect of the addition of NaOH and KOH on the reactivity of partially hydrated calcium hydroxide. Working with putrefied biological material is, to say the least, challenging from an olfactory standpoint, and in a study on the aroma of truffles, Guareschi mentions that the air coming from tissues in putrefaction could be made odorless, except for a mild “truffle flavor”, when passed through freshly prepared soda lime [73]. The Guareschi mask (Figure 6) was eventually turned down by the Italian army since it was too heavy compared to the Ciamician–Pesci swab-type mask, the inefficiency of which became, however, tragically evident when the large-scale use of gas started on the Italian front at the end of 1917. The Guareschi mask was reconsidered, but in early 1918, Guareschi’s health was declining rapidly, while his son had been severely wounded at the front, and neither of them could go to Rome and promote the use of the mask (Supporting Information File 1, tenth paragraph). Even though the Guareschi mask was never developed, the American Chemical Warfare Service acknowledged the priority of Guareschi in the development of the soda lime gas mask, securing his fame in military history [9]. During the war, different gas masks were developed. The German one was based on activated charcoal and hexamethylenetetramine and was developed by Haber and Willstätter [74], while the Russian version was developed by Zelinsky and used charcoal to absorb poisonous gases [75]. According to Willstätter, the Anglo–French mask was originally a copy of the German one [28], later replaced with the soda lime gas mask that Guareschi had originally developed [9].

**Figure 7:**
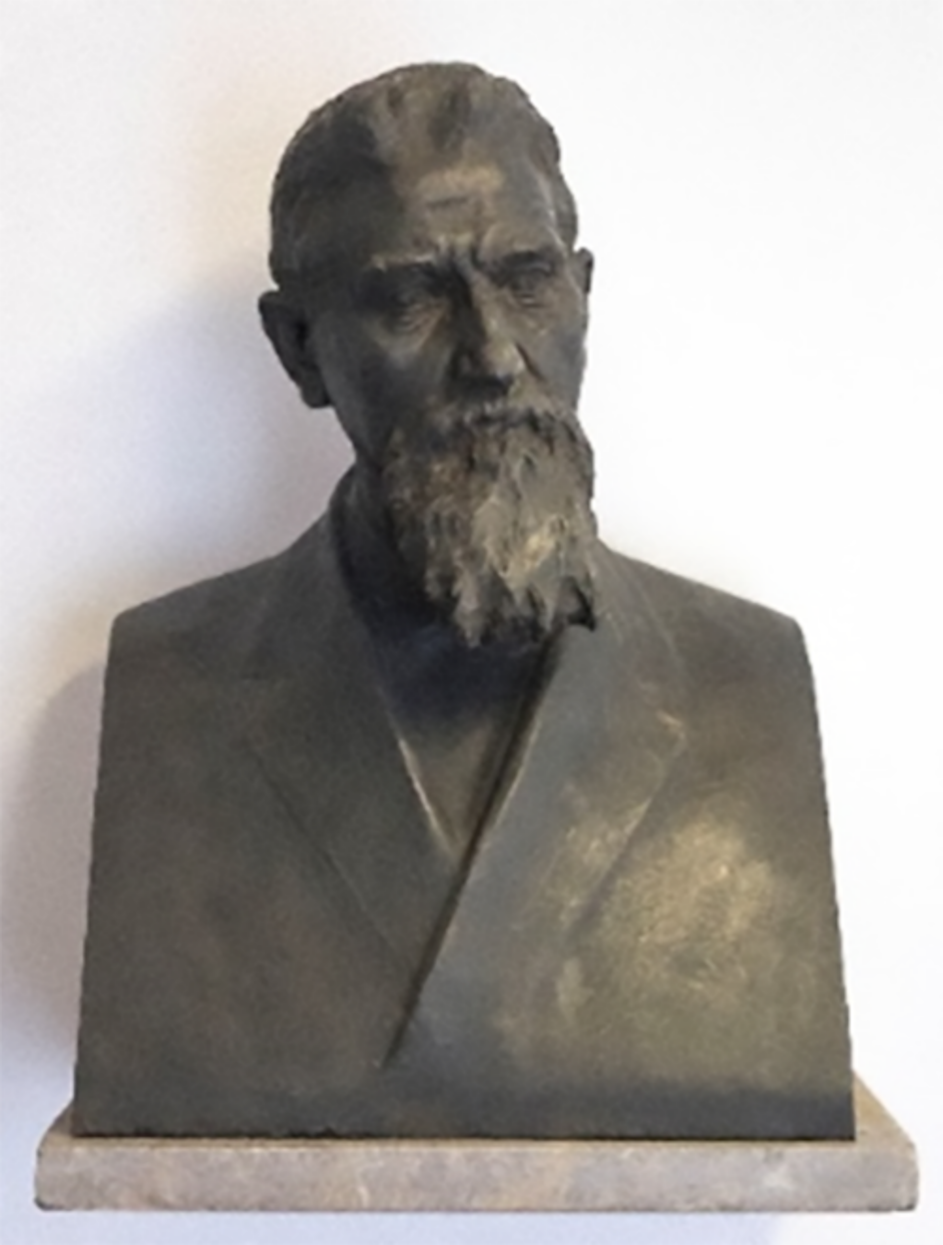
Guareschi’s bust at the Dipartimento di Scienza e Tecnologia del Farmaco of Turin University. Permission of reproduction from Dipartimento di Scienza e Tecnologia del Farmaco, Università di Torino is gratefully acknowledged. This content is not subject to CC-BY 4.0.

On the forecast of a possible general famine, Guareschi was called to chair the National Committee for the improvement of the agricultural practices. It is surprising that a chemist was put into this position, and a possible reason could be Gureschi’s multidisciplinary expertise, as testified by the role he played in all the three scientific academies of Turin. Apart for his commitments with the Academy of Sciences, he also headed the one of Medicine in 1903–1905, next serving as vice president of the one of Agriculture in 1917–1918. Guareschi’s first task was to revise the agricultural and nutritional treatises popular in Italy. In doing so, he noticed the almost complete lack of chemical considerations in agricultural manuals and spotted blatant errors of significant nutritional relevance, such as the location of gluten exclusively in the tegument of wheat. Guareschi could be considered the initiator of what would then be called by the Fascist regime “the war of wheat”. At the turn of the 19th century, nutritional studies were biased in a way that considered wheat as the most suitable grain for the "Caucasian race", to cite a contemporary view at that time. This concept was vividly expressed by William Crookes (1832–1919), the discoverer of thallium and of the eponymous tube, in the inaugural speech he gave in 1898 at the annual meeting of the British Society for the Progress of Science [76]. This historical speech is often quoted for the vehemence by which it advocates the urgency of developing ways to fix atmospheric nitrogen, alas in a biased and discriminating way. Crookes expressed his claims as a "Cahier des Doléances" on the state of agriculture in Europe and on his perceived danger that, unless the fertility of European soils is increased, “the great Caucasian race will cease to be the most advanced, and will be wiped away by races whose life does not depend on wheat and bread”. Reflecting on the Italian situation, Guareschi urged the Italian Government to increase the cultivation of wheat at the expenses of the, for him outrageously large, area dedicated to grapes, also investing in variety improvement. This was then achieved twenty years later by Nazareno Strampelli (1866–1942), the forerunner of the Green Revolution of the late 1960s [77]. Guareschi supported a dietary regime low on meat and animal proteins and rich in raw vegetables, with a particular emphasis on the replacement of white bread with whole bread. He approached the comparison of these two types of bread with the same gigantic spirit J. S. Bach approached the humble Basso di Ruggero for his Goldberg variations: 56 pages, 11 numeric tables, and a final table summarizing all the nutritional comparative studies on white and whole bread carried out on different animal species [78]. The dietary suggestions of Guareschi were considered too radical, to the point that, when he collapsed in his department while chairing an academic promotion committee, eventually dying one week later (on the night of June 20, 1918), it was suggested that his dietary regime had made him weak, ultimately causing his death. Four years later, a public subscription made it possible to honor Guareschi with a bust, wrought by the sculptor Giovanni Cellini from the bronze of captured Austrian cannons, a paradoxical material for a pacifist like him (Figure 7). Remarkably, one of the subscribers was Edmund Oscar von Lippmann (1857–1940), famous in the context of sugar, who had shared Guareschi’s interest for the early history of chemistry and for the work of Marcellin Berthelot. Guareschi’s body was cremated, a rather unusual event for those times but common within the Freemasonry community. Since his daughter Maria had received catholic obsequies, we presume that the body of Guareschi was cremated because of his own precise will. Until 1983, cremation was forbidden by the Catholic Church. Cremation was indeed rare and stirring, to the point that in 1947, when the ashes of the Nobel laureate Luigi Pirandello, who had died in 1936 in Rome, were buried in his native Agrigento, they had to be put into a coffin to simulate a mainstream funeral and avoid a scandal [79].

The scientific production of Guareschi spans 47 years and comprises almost 500 entries, only ≈150 of which belong to the realm of organic chemistry. Most of the remaining entries deal with the history and the technological applications of chemistry and with nutrition. The overall idea is that Guareschi was an organic chemist by profession and a disseminator of chemical knowledge by vocation, in sharp contrast to the view of the Cannizzaro school, which considered the critical analysis of literature a “waste of time” compared to the experimental work in the laboratory [65]. In an assay on chemistry and art, Guareschi commented that the pace of scientific advancements of his times was too fast, and historical studies were a way to make a break, look back at the travelled way, and marvel [80].

## Conclusion

Guareschi’s technical studies, such as those on the elemental analysis of gases and the determination of hydration in salts, were popular in his times but have now been made obsolete by instrumental advances, just like his sodium amalgam reduction of amides to aldehydes has disappeared from the organic chemistry armamentarium of reactions. His studies on the technology of Renaissances inks and ancient dyes were among the first scientific studies in an area that spectroscopic analyses have modernized. Biochemical studies have provided a solid foundation to his nutritional emphasis on raw food but, to the benefit of wine lovers, Italy has not replaced the cultivation of grapes with the one of wheat. Guareschi’s huge historical work on the Italian chemistry in the second half of the 18th century substantially failed to make scientists like Marsigli, Melloni, Basso, and Malaguti household names in the history of chemistry and, to Guareschi’s dismay, we still refer to the gas pressure law as the Gay–Lussac and not as the Volta law. On the other hand, Selmi is now recognized along with Graham as the initiator of the study of colloids, and Avogadro occupies a preeminent position in the Pantheon of founders of modern chemistry. For a sort of retaliation, the broadness of interests of Guareschi was followed, in the organic chemistry landscape of Turin, by the sort of behavior that led Giacomo Ponzio (1870–1945) and Luigi Mascarelli (1877–1941) to publish 124 articles on a single relatively minor class of organic compounds, the dioximes (Ponzio, Supporting Information File 1, eleventh paragraph) and to classify the preparation and physical properties of 5,000 biphenyl derivatives (Mascarelli, Supporting Information File 1, twelfth paragraph). Nowadays, Guareschi is remembered for his pyridine synthesis, for the wet chamber for parchment restauration, and for the soda lime gas mask, three achievements that in the current “zonated” scientific environment are surprising to be attributed to a single person. His isolation of succinic acid from muscle tissues has historical relevance, but the discovery of Guareschi imides, a class of compounds that liberate hydrocarbons upon treatment with water and air, goes beyond a simple curiosity. We hope this monograph on Guareschi will inspire additional research on the “1897 reaction”, identifying other systems that exhibit the remarkable properties of Guareschi imides, and eventually exploiting the easy generation of carbon radicals under the green conditions of the reaction to develop new radical processes of synthetic relevance.

## Supporting Information

File 1 Additional notes.
