# Supplementary material for: Icilio Guareschi and his amazing “1897 reaction”
Source: Beilstein J Org Chem. 2021 May 25;17:1335–51. doi: 10.3762/bjoc.17.93 (PMC8182683; doi:10.3762/bjoc.17.93)
Supplement: File 1 — Additional notes. [file Beilstein_J_Org_Chem-17-1335-s001.pdf]

BEILSTEIN JOURNAL OF ORGANIC CHEMISTRY

Supporting Information

for

Icilio Guareschi and his amazing “1897 reaction”

Gian Cesare Tron, Alberto Minassi, Giovanni Sorba, Mara Fausone and Giovanni Appendino

Beilstein J. Org. Chem. **2021,** *17,* 1335–1351. doi:10.3762/bjoc.17.93

Additional notes

License and Terms: This is a supporting information file under the terms of the Creative Commons Attribution License (https://creativecommons.org/licenses/by/4.0). Please note that the reuse, redistribution and reproduction in particular requires that the author(s) and source are credited and that individual graphics may be subject to special legal provisions.
The license is subject to the *Beilstein Journal of Organic Chemistry* terms and conditions: (https://www.beilstein-journals.org/bjoc/terms)

1. Schiff's parsimony was legendary. Paternò recalls that Schiff used to travel from Florence to Rome with a suitcase containing only a shirt and a pair of shoes that he proudly carried without a porter, claiming that there was no need to pay another person to do something he could do by himself (de Condé Paternò di Sessa, M. E. Paternò di Sessa. *Emanuele Paternò di Sessa. Dall'esilio alla fama scientifica: Scienza e Politica fra il XIX ed il XX secolo vissute da un protagonista.* Gangemi, Palermo, 2018, p165). Schiff was rumored to eat mostly dried chestnuts, but enjoyed excellent health until he was run over by a cart in 1899 (Guareschi, I. *Atti R. Acc. Sci Torino*, **1917**, *53*, 333–352). He never fully recovered from this accident, reminiscent of the fatal one of Pierre Curie in Paris seven years later. It was such a common belief that Schiff lived in dire poverty that, to celebrate his 70th birthday, a subscription to support him raised the equivalent of 20,000 € in the Italian and European chemical community. Schiff did not like the initiative and destined the sum to an annual prize for the best thesis in chemistry. When Schiff passed away, it was discovered that, thanks to his parsimonious life, he had accumulated a small fortune, 70% of which, amounting to over 1.5 million €, he destined in his will to the establishment of a Schiff Foundation to support disabled workers. The Foundation was extinguished only in 1983 (Costa, L.; Guarna, A. *Rendiconti della Accademia Nazionale delle Scienze detta dei XL Memorie di Scienze Fisiche e Naturali, serie V* **2011**, *35*, 153–164). Regarding the temperamental behavior of Schiff, suffice to mention the case of the physicist Antonio Roiti (1843–1921). Faced with the problem of the precise determination of some electrical constants, Roiti was working at night to avoid the interference of trams. He could do nothing, however, with the interferences created ad hoc by Schiff, who was moving heavy metallic containers in a nearby corridor to disturb the measurements. In his final report, Roiti wrote that his results were precise "despite the malice of one of my colleagues" (Costa, M.; Fontani, M. *Microstoria Rivista toscana di Storia locale* **2007**, *52*, 62).

2. Emanuele Paternò was remarkable not only for the discovery of the eponymous carbonylolefin photocycloaddition, but also for his clear elaboration of the tetrahedral nature of carbon, a concept that he developed while studying the isomerism of trichloroethane six years before the publication of the works by van't Hoff and Le Bel. 22-year-old Paternò published his finding in a local journal (Intorno all'azione del percloruro di fosforo sul clorale, *Giornale di Scienze Naturali ed Economiche*, **1869**, vol. V) and then sent it to Adolf Lieben in Turin for a possible publication in Berichte. Lieben's answer was trenchant: "Your theory of tetrahedral carbon crosses the Rubicon that separates the licit speculations on how atoms combine from the illicit speculations on how atoms position in space. By rising up in space looking for atoms, you lose ground under your feet." Frustrated, Paternò abandoned the study of stereochemistry, something he regretted all his life, however without ever claiming priority for the discovery of the tetrahedral nature of carbon, whose first formulation he ascribed to Kekulé. Curiously, Kolbe commented van't Hoff 's theory of tetrahedral carbon in a way reminiscent to Lieben's comments to Paternò: According to Kolbe, van't Hoff, rather than experimental investigations, has considered more comfortable to mount Pegasus and to proclaim in his "La chimie dans l'espace" how the atoms appear to him to be arranged in space when he is on the chemical mount Parnassus which he has reached by bold flight (Kolbe, H. *J. Prakt. Chem.* **1877**, *15*, 474). Writing in the late 30s, Willstätter explained in a similar way the aversion of Haber to organic chemistry, whose PhD thesis was on the chemistry of safrole: Structural chemistry is important and beautiful as long as we do not expect the formulas to express too much. A chemist who tries to explore the concepts of affinity and valence more searchingly with the tools of organic chemistry, however, runs the risk of uncertainty, inaccuracy, and sloppiness (R. Willstätter, *Aus meinem Leben* (Ed. A. Stoll), Verlag Chemie, Weinheim, 1949; English translation: From My Life, Benjamin, New York, 1965., Chapter 10, p 409). The political career of Paternò was remarkable. He was Rector of the University and then major of Palermo, moving next to Rome in 1892 as a

senator and to succeed Cannizzaro at the chair of analytical chemistry. In Rome, Paternò served as a vice president of the Italian Senate from 1904 to 1919 and also held important position in the Italian Freemasonry. Paternò kept all his life the letter he had received from Lieben, which is reproduced in a book that collects part of the manuscripts of the Paternò fund of the Italian Academy of Science (Accademia dei XL), an institution that Paternò chaired from 1921 to1932 (de Condé Paternò di Sessa, M. E. Paternò di Sessa. *Emanuele Paternò di Sessa. Dall'esilio alla fama scientifica: Scienza e Politica fra il XIX ed il XX secolo vissute da un protagonista*. Gangemi, Palermo, 2018).

3. Maria Guareschi (1875–1909) helped her father with translations from and into English and German. Her translation of the textbook Grundlage der Chemie by Sophus Mads Jørgensen, Werner's rival of coordination chemistry, was published in 1904 (for an account of the Werner–Jørgensen controversy. see: Kauffman, G. B. *J. Chem. Ed.* **1959**, *36*, 521–527). Jørgensen, who was fluent in Italian, considered the translation outstanding (eccellente). When Maria prematurely passed away in Naples, Guareschi received condolence letters from some major European organic chemists, including Willstätter, Moissan, Eugler, as well as from Berthelot's son. Guareschi was an outspoken supporter of the role of women in academia and research. He maintained a female collaborator in his laboratory (Maria Clotilde Bianchi) until she was forced to enroll as a military pharmacist in 1915, and a photography of Guareschi's group in the early 1900 shows three female members. Women were very rare in chemistry laboratory those years. Emil Fischer himself was allowed to host women in his Institute only in 1909. Until then, Lise Meitner could only work in Otto Hans's laboratory and was not allowed into the rest of the Institute, which she was accessing from a side door. In the more liberal France, women were allowed in laboratories but, as testified by the early work of Marie Curie, could not receive a salary as laboratory assistants.

4. Berthelot is buried at the Pantheon in Paris, and it was suggested to put this inscription on his tomb: "Ci-gît Marcelin Berthelot, dans la seule place qu'il n'a pas sollicitée" (Here lies Marcelin Berthelot, in the only place he has never claimed, de Condé Paternò di Sessa, M. E. Paternò di Sessa. *Emanuele Paternò di Sessa. Dall'esilio alla fama scientifica: Scienza e Politica fra il XIX ed il XX secolo vissute da un protagonista.* Gangemi, Palermo, 2018, p 153).

5. Selmi had been given death penalty for his involvements in the revolution of 1848. He therefore fled to Turin, where he was hosted in the laboratory of Ascanio Sobrero, the most important Piedmontese chemist of the time, and he also enjoyed the friendship of the powerful politician Camillo Cavour. Selmi promoted the surge of the Duchy of Modena that eventually led to its annexation to Piedmont in 1859 and was honored, along with the composer Giuseppe Verdi, with the honor citizenship of Turin, eventually heading the Ministry of Education of the Italian Kingdom. Selmi started the publication of a comprehensive and monumental chemistry encyclopedia (Enciclopedia di Chimica), a project interrupted by his death and terminated by Guareschi. In 1913, Guareschi started the publication of an updated version, named Nuova Enciclopedia della Chimica which, after his death, was terminated by his son-in-law Felice Garelli (1869–1936).

6. Sobrero lost an eye during his research on nitroglycerine in his laboratory in via Po 18. The chemistry Institute was located in the courtyard of the building and hosted a magnificent amphitheater. It was nicely decorated and was considered one of the best lecture rooms in Turin, capable of hosting up to 500 students. It survived Sobrero's experiments with nitroglycerine but was demolished by a controversial decision in the 20s of the past century. Sobrero stored ≈300 mL of his original preparation of nitroglycerine in a sealed flask whose conservation posed embarrassing problems. Nobel was interested in this heirloom and, after

washing its contents with a bicarbonate solution to remove any nitric acid decomposition product, Sobrero donated the refilled flask to the Nobel plant in Avigliana, writing a document where he testified that the flask and the contents were those of his original studies. The flask, with its contents replaced by a nonexplosive liquid, is now stored at the Nobel Museum in Sanremo (https://villanobel.it/, Garbarino, G.. *Alla Scoperta di Ascanio Sobrero*. Centro Stampa Cavallermaggiore, 1995. p193–195).

7. Angelo Mosso (1846–1910) is considered the father of studies on high-altitude physiology, an area he investigated in a laboratory built at an altitude of over 4,000 m on Monte Rosa, the second-highest mountain in the Alps. Diagnosed with a severe form of tabes dorsalis, he had to abandon active biomedical research. Advised to pursue open-air rather than laboratory activities, he reinvented himself as an archaeologist, giving important contributions to expeditions to Crete and Southern Italy. His “human circulation balance” is nowadays considered the first neuroimaging technique ever produced (Sandrone, A. *Brain* **2014**, *137*, 621–633). Mosso and Guareschi first met in Florence, where Guareschi was working in Hugo Schiff’s laboratory and Mosso in the physiology laboratory of Hugo’s elder brother Moritz (1823–1896), famous for his studies on thyroid.

8. A curious application of the bromine color reaction was the identification of the remains of important persons in archaeological multiple burials. In ancient times, important people were buried wrapped in clothes dyed with Tyrian purple, a bromine-containing bisindole. When Saint Ambrose’s tomb in his eponymous church in Milan was opened in 1871, three skeletons were discovered in it, and the saint was identified a few years later by Roberto Giorgio Lepetit from the presence of bromine in his bones (Sironi, V. A. *Le Officine della salute*. Laterza, Bari, 1992, p 49). The identification was important because Ambrose is the

protector Saint of Milan, and his church is where the first act of Verdi's I Lombardi alla Prima Crociata is staged.

9. The case of the Eugenio Elia Levi (1883–1917), one of the major Italian mathematicians of those times, is emblematic. In 1915, his application to join the army at the age of 32 was turned down since he was shorter than 154 cm, the minimum height to become a soldier. Unhappy, Levi appealed, and on account of a modification of the rules of enrolment, he could later enter the army and go to the front. However, when a law preventing public officers to volunteer for the army was implemented, he was sent back to the University of Genoa, which had classified his participation to teaching activities as indispensable. Claiming that the law could not be applied retroactively, Levi appealed again, and eventually managed to reach the front. He died on October 28, 1917 hit by a bullet in the head (Guerraggio, A. *La Scienza in Trincea*, Raffaello Cortina Editore, 2015, p 52). Levi's enthusiasm to participate in the war is reminiscent of the one of Otto Warburg, who fought on both the Western and the Eastern fronts. It took Albert Einstein, a family friend, to convince him that he was more useful to Germany in the laboratory than at the front. Eventually, he applied to be released from active service and was discharged in the summer of 1918, in the last months of the war (Kippenal, W. H.; Bounds, P. L.; Dang, C. V. *Nature Rev. Cancer* **2011**, *11*, 325–337).

10. Guareschi had two sons (Giacinto and Pietro) and one daughter (Paolina) from his second wife. Giacinto (1882–1976) pursued an academic career as a mathematician. His son Marco (1922–1945) was active in the Italian resistance movement and died in the Mauthausen concentration camp one month before the end of the war. In late 1917, 70-year-old and already ill Guareschi went to the front to rescue his severely wounded son Pietro, liberating him from captivity. We have been unable to clarify how Guareschi managed to do so, but also Le Chatelier had a son wounded on the Italian front and, thanks to the

agency of Paternò, he came with his wife from France to the Eastern Alps to assist him (de Condé Paternò di Sessa, M. E. Paternò di Sessa. *Emanuele Paternò di Sessa. Dall'esilio alla fama scientifica: Scienza e Politica fra il XIX ed il XX secolo vissute da un protagonista.* Gangemi, Palermo, 2018, p154).

11. Ponzio was storing all the dioximes prepared in his laboratories in a library of flasks (still preserved) that a technician had to label and update. For unclear reasons, a wall was built between the Pharmacy and the Chemistry Departments at the time of the Ponzio-Mascarelli diarchy.

12. Mascarelli succeeded Guareschi as the chair or pharmaceutical chemistry and toxicology. Of Piedmontese origin (Bra), he came from the Ciamician school in Bologna. Mascarelli was held in high esteem by Ciamician and was given the task to order his lessons into a textbook. He is remembered for his remarkable eponimic reaction of transformation of biphenyls into fluorenes and is considered one of the founders of the chemistry of these compounds as well of the application of chlorinated diphenyls as electrical insulators. While working in Bologna, he reported in 1907 the discovery of aryliodonium salts (Mascarelli, L. *Atti della R. Acc. dei Lincei. Rendiconti, cl. di Scienze Fisiche, Mat. e Naturali* **1907**, *16*, 562–567), a reaction rediscovered three decades later. The ultimate attribution to Mascarelli of the discovery of these compounds led to the recall of a string of patents filed on aryliodonium salts.
